# Biotechnological Applications of Eggshell: Recent Advances

**DOI:** 10.3389/fbioe.2021.675364

**Published:** 2021-07-06

**Authors:** Tamer A. E. Ahmed, Ling Wu, Manar Younes, Maxwell Hincke

**Affiliations:** ^1^Department of Cellular and Molecular Medicine, Faculty of Medicine, University of Ottawa, Ottawa, ON, Canada; ^2^School of Nutrition Sciences, Faculty of Health Sciences, University of Ottawa, Ottawa, ON, Canada; ^3^Department of Innovation in Medical Education, Faculty of Medicine, University of Ottawa, Ottawa, ON, Canada

**Keywords:** eggshell, patents, biotechnological applications, biomedical, chemical, engineering, environmental, technologies

## Abstract

The eggshell (ES) provides protection against pathogenic and physical insults while supplying essential metabolic and nutritional needs for the growing avian embryo. It is constituted mainly of calcium carbonate arranged as calcite crystals. The global chicken egg production in 2018 was over 76.7 million metric tons. In industrialized countries, about 30% of eggs are processed at breaker plants that produce liquid egg products and large quantities of solid ES waste. ES waste is utilized for a variety of low-value applications, or alternatively is disposed in landfill with associated economic and environmental burdens. The number of patents pertaining to ES applications has increased dramatically in recent years; of 673 patents granted in the last century, 536 (80%) were published in the last two decades. This review provides a snapshot of the most recent patents published between 2015 and 2020, with emphasis on different biotechnological applications of ES waste, and summarizes applications for biomedical, chemical, engineering, and environmental technologies. Biomedical technologies include the production of calcium lactate, calcium phosphate, and health-promoting products, while chemical technologies include plant growth promoters, food processing and production, and biodiesel oil catalysis along with active calcium, carbon, soluble proteins, organic calcium, and ultrafine calcium carbonate sources. Engineering technologies address material engineering and nanoparticle production, while environmental technologies pertain to production of biomass, solubilization of sludge as well as production of magnetic ES adsorbents and adsorption of heavy metals, organics, total nitrogen and fluoride, soil pollutants, and radioactive compounds. Although the number of ES-based patents has exponentially increased in the last decade, exploration of innovative top-down approaches and ES development as a physical platform are new endeavors that are expected to further increase the upscaling of ES waste exploitation.

## Introduction

### Eggshell Function and Composition

The calcareous egg is a reproductive vehicle for all avian and most reptilian species. The avian egg represents the most complex amniotic egg in oviparous vertebrates (Hincke et al., 2012). The eggshell (ES) has evolved to offer protection against physical and pathogenic insults while providing metabolic and nutritional needs for the growing embryo (Rose and Hincke, 2009; Hincke et al., 2012; Stapane et al., 2019). The partial dissolution and associated thinning of ES during fertilized egg incubation provides calcium for calcium-phosphate mineralization of the growing embryonic chick skeletal system, along with facilitating the chick hatching/pipping at the end of incubation (Athanasiadou et al., 2018).

**FIGURE 1:**
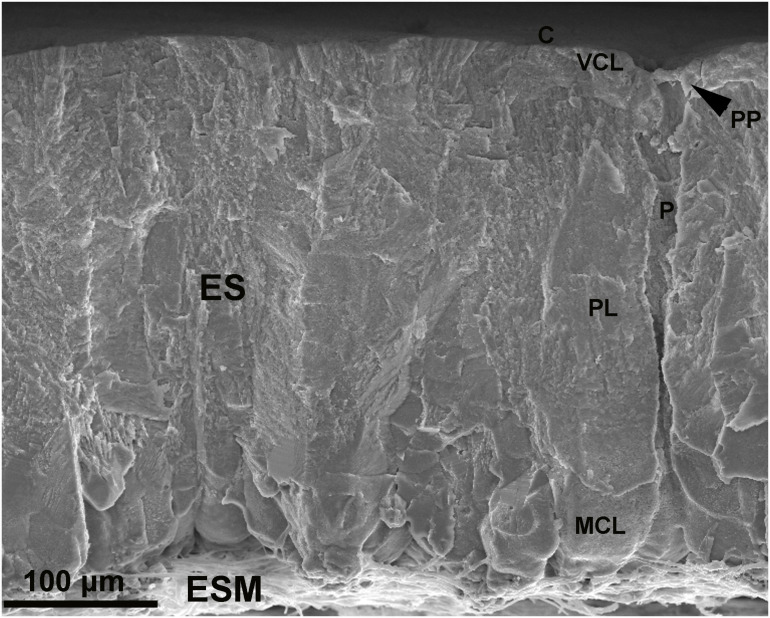
Morphology of the eggshell and eggshell membranes revealed by scanning electron microscopy (SEM). Cross-fractured eggshell reveals the calcified eggshell (ES), constituted of cuticle (C), vertical crystal layer (VCL), palisade layer (PL), and mammillary cone layer (MCL); with the associated eggshell membranes (ESM). Respiratory pore (P); pore plug (PP). (Nano Imaging Facility, Carleton University, Ottawa, Canada).

The chicken ES is constituted of calcium-carbonate (CaCO_3_) mineral in the form of calcite (∼95%) and organic material/matrix (∼3.5%; Athanasiadou et al., 2018). The organic matrix consists of proteins and proteoglycans that interact with the forming mineral phase to confer specific microstructural and mechanical properties to ES (Hincke et al., 2012). ES-specific matrix proteins direct the protective functions of ES during avian reproduction, through regulation of ES mineralization (physical protection) and antimicrobial defense (chemical protection; Rose and Hincke, 2009). The ES presents a multi-laminated resilient structure consisting (from inner to outer) of eggshell membranes (ESM), mammillary cone layer, palisade layer, and cuticle (Nys et al., 2004; Hincke et al., 2012; Figure 1).

### Egg Market

Chicken eggs are a vital part of the daily human diet worldwide and serve as a cost-effective and high-quality nutritious food (Ahmed et al., 2019a). Global egg production in 2018 was over 76.7 million metric tons (Shahbandeh, 2020). The five leading egg-producing countries are China, United States, Indonesia, India, and Mexico which together contributed approximately 63% of total global egg production (∼1,652 billion eggs, 99 million tonnes) in 2019 (FAO, 2020).

In industrialized countries, about 30% of shell eggs are redirected to breaker processing plants to produce liquid egg products, and are not consumed as shell eggs (Cordeiro and Hincke, 2011; Ahmed et al., 2019a,b). ES and the associated ESM represent approximately 10% of egg weight and are therefore produced in large quantities as a byproduct of the egg-processing industries, estimated at 2.3 million tons worldwide (Hincke et al., 2012; Laca et al., 2017). The solid ES residues generated by these operations are often discarded in landfill sites without any pre-treatment (Cordeiro and Hincke, 2011). Decomposition of ES, ESM and the associated residual egg white (EW) leads to the release of ammonia (NH_3_) and hydrogen sulfide (H_2_S) with an offensive odor, which attracts rodents and insects (Owuamanam and Cree, 2020). Industrial egg producers must dispose ES waste properly in landfill sites according to local environmental regulations; however, this discards the potentially valuable calcium carbonate and bioactive protein constituents. Furthermore, the costs associated with landfill disposal of egg waste are projected to climb with inflation (Ahmed et al., 2019a).

### Eggshell Waste and Disposal

Globally, the total amount of ES waste was about 2.3 million tonnes in 2018 (Hincke et al., 2012; Laca et al., 2017; Ahmed et al., 2019a). China produced 458,448 million eggs in 2018 (FAO, 2020), of which 137,534 million eggs were processed in breaking plants and generating 825,204 tonnes of mineral-rich ES waste. In the United States, ES waste generated by food industries is ranked 15th on the Environmental Protection Agency list of waste that creates pollution problems, as its disposition in landfill leads to environmental issues. The cost of ES waste disposal to landfill by one American breaker plant can be approximately $100,000 USD annually (Owuamanam and Cree, 2020). In Canada, all egg producers together pay about $0.8–1.6 million USD to dispose of ES waste (Canadian Food Innovators, 2015). In Europe, small or medium-sized egg enterprises spend $127,000 USD per year for ES waste disposal (Shellbrane, 2012). More specifically, Just Egg Ltd. (Chilled foods, United Kingdom), which produces 17.7 tons of ES waste annually, claims that it spends $66,000 USD per year to dispose of this material (Shearman, 2016; Just Egg Ltd., 2020).

### Eggshell Waste Added-Value Applications

**FIGURE 2:**
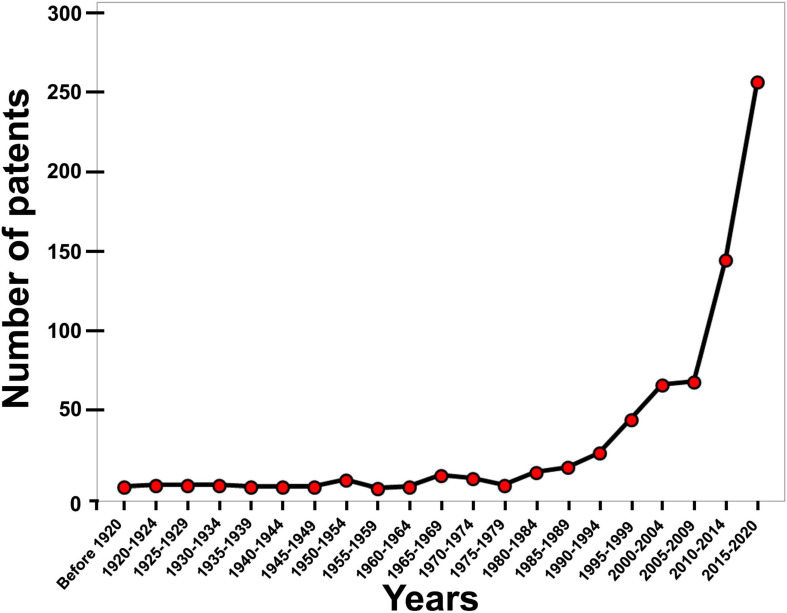
Chronology for patents describing ES-related devices and methods in the last century. An exponential increase in patent numbers in the last two decades is apparent (Data derived from the Google patent research engine).

**FIGURE 3:**
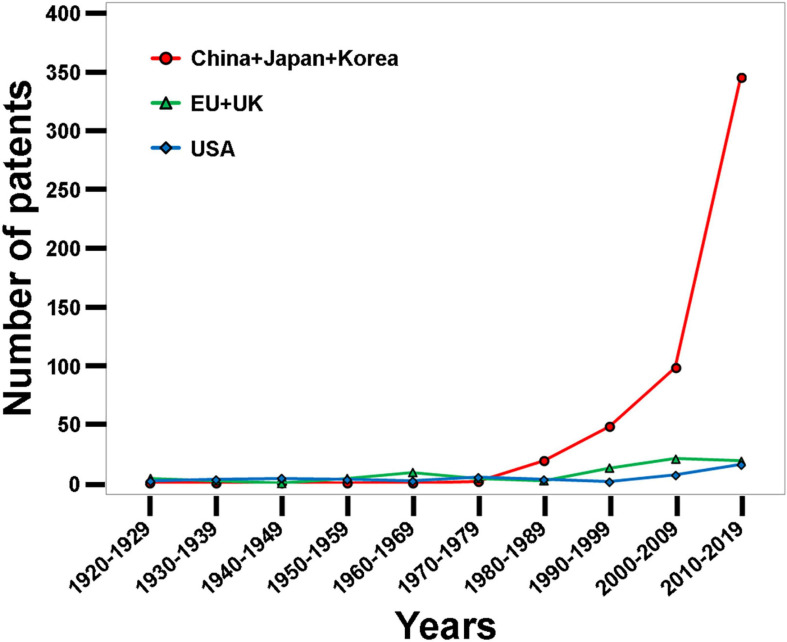
Geographic distribution of ES patents in the last century amongst different countries, showing the recent dramatic output from Asian countries (China, Japan, Korea, and Taiwan), which is responsible for the observed exponential increase in patent numbers over the last two decades (Data derived from the Google patent research engine).

**FIGURE 4:**
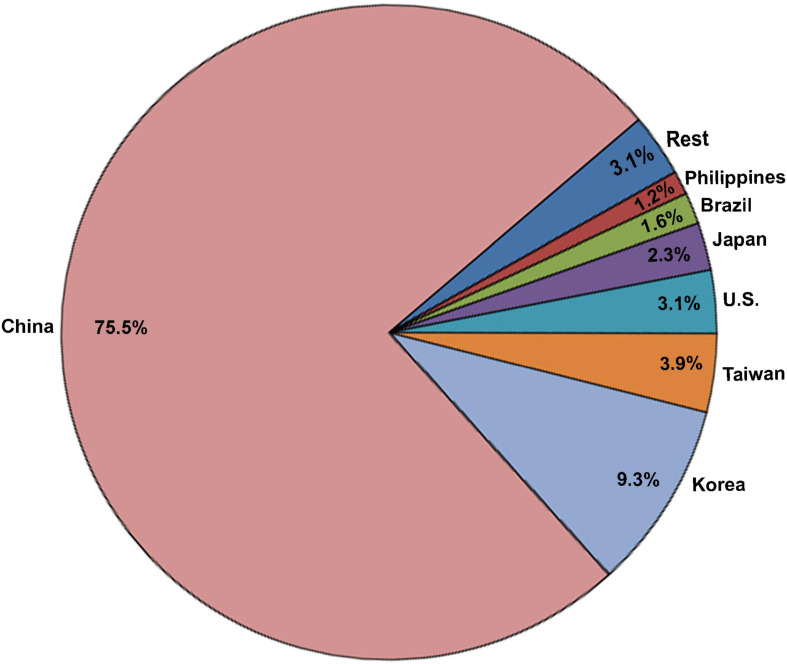
Pie chart showing the geographic distribution of ES-related patents between 2015 and 2020 amongst different countries (Data derived from the Google patent research engine).

Eggshell waste is rich in bioactive compounds; therefore, there is increasing investment in research to develop value-added ES-derived products with commercial applications (Cordeiro and Hincke, 2011). A large number of applications to generate value-added products from ES waste have been patented; their number has exponentially increased during the last two decades (Figures 2–4).

In 2012, the European Commission and the Seventh Framework Program (FP7) funded a 2-year project entitled “Separating ES and its membrane to turn ES waste into valuable source materials,” with a budget of over 1.5 million € (Shellbrane, 2012), to help mitigate the environmental problem of ES waste (Shellbrane, 2012, 2019). The Consortium developed a prototype capable of processing 60 kg/h of ES waste (about 10,000 eggs/hr), producing high quality ESM and ES. The prototype consists of three modules: separation, disinfection, and drying (CORDIS). A similar goal was implemented in Canada including funded projects for the repurposing of ES waste protein as potential medical and health-care spinoffs (Cordeiro and Hincke, 2011; Poultry Innovations Conference, 2013) and development of ES powder as a functional food ingredient (Canadian Food Innovators, 2015). The valorization of ES biowaste will improve both the environment and the balance sheets of egg producers.

### Potential Markets for Eggshell Waste

The chicken ES consists of calcium carbonate (CaCO_3_) mineral in the form of calcite (∼95%) and organic material/matrix proteins (∼3.5%; Hincke et al., 2012). The global market for calcium carbonate (ground and precipitated) is projected to reach USD 28.98 billion by the end of 2025, growing at a compound annual rate of 5.78% (ReportLinker, 2020), without taking it into account as a substantial market for high calcium content ES waste. ES waste could be valorized as a partial replacement for commercial products such as limestone, pure calcium carbonate, soil conditioners, biomaterials, food additives or supplements, cosmetic or pharmaceuticals base, catalyst, and wastewater purifiers (Waheed et al., 2019; Mignardi et al., 2020; Owuamanam and Cree, 2020).

In 2019, more than 50% of geologic calcium carbonate (limestone) was used for steel manufacturing, and the remaining market was shared by building and construction, water treatment, agriculture, paper and pulp, plastics, and paint (Cree and Rutter, 2015; Limestone, 2019; Lewicka et al., 2020). The price of commercially available calcium carbonate limestone powder is around $ 200–350 USD/metric ton (Katsuyama et al., 2005). On average, about 88∼270 kg limestone is used for the production of every ton of crude steel. According to the World Steel Association, 1870 million metric tons of steel were manufactured in 2019 (Mordor Intelligence, 2020), which requires limestone with a value of at least $33 Bn USD. ES waste could partially replace limestone use in the steel industry, thus opening a substantial market. In addition, the building and construction industry is the second largest market for limestone consumption, in which Ordinary Portland Cement (OPC) is a precursor for concrete. The world production of OPC is predicted to exceed 5 billion metric tons by 2030 (Imbabi et al., 2012), which consumes limestone reserves. This depletion could be mitigated via the use of ES waste.

Another potential market for ES waste is paper manufacturing. The paper industry uses limestone-based raw material to produce fillers and coating pigments, such as CaCO_3_ pigment for printing paper and board (Gaber, 2018). In Europe, around 90 million tons of paper and board were produced in 2019, of which more than half were directed toward packaging (container board, carton board, and wrapping, etc.), in addition to graphic papers (newsprint, printing, and writing, etc.) and other uses (e.g., tissue; Limestone, 2019). In the largest European paper plants, around 385 kg of fillers (CaCO_3_) and coating pigments (CaCO_3_ and kaolin) are used for every ton of paper production. These paper plants produced over 1,200 million tons of paper in 2012 that required approximately 47,300 tons of CaCO_3_ and kaolin, which is another market for ES waste (Lewicka et al., 2020). In addition, ES showed potential as a replacement for limestone to prepare hydroxyapatite (HAP) utilized for the sustainable treatment of toxic metal-polluted water. The transformation of ES into ES-based hydroxyapatite (ESHAP) on an industrial scale was claimed to create an investment return at least 5 times greater than the cost of conventional disposal expenses (Mignardi et al., 2020).

Calcium compounds are part of the broad applications market for calcium carbonate, calcium citrate, calcium phosphate, and calcium chloride, etc. in the biomedical, nutritional and pharmaceutical sectors. The global calcium citrate and calcium phosphate markets are anticipated to reach USD 900 million by 2025 (Ahuja and Rawat, 2019). Commercial calcium supplements are commonly produced from dried shrimp, fish, oyster shells, coral, and algae (Xu et al., 2020), the use of which might not be ecologically sustainable going forward. ES is an acceptable source of calcium supplement for animal feed and human health (Schaafsma et al., 2000; Lewicka et al., 2020). Moreover, the calcium extraction efficiency from oyster shell waste was shown to be lower than that from ES (Chakraborty and Gaonkar, 2016). Over the past decades, different methods have been developed to obtain calcium citrate or calcium chloride from ES (Waheed et al., 2019), making it an attractive raw material for the supplement market.

Calcium oxide (CaO), also known as lime or quicklime, can be derived from the calcination of ES. CaO is utilized as a neutralizing agent in the agricultural, petrochemical, cosmetics, pharmaceutical, animal feed and tanning industries, or as a filler for aluminum, plastics, cement, glass, and paper [Other Industrial Consumers & Manufacturing – EuLA: European Lime Association. EuLA (2020)]. These sectors provide a considerable market for ES upcycling. Quicklime was valued at about $124 per ton in 2019 [USGS Online Publications Directory| Lime. USGS (2020)]. Different type of glasses, such as household and crystal glass, flat glass, and typical container glass, contain approximately 5 to 12% CaO (Lewicka et al., 2020), and their production is a potential market for ES.

### Commercially-Available Eggshell-Based Products

The calcium supplement market is an attractive sector for applications of ES waste. Bone Health Original^TM^ capsules target bone heath, and advertise ES as an easily digestible, superior source of calcium (Bone Health Original). Similarly, OVOCET^®^ is a product based on ES, as a supplementary source of highly absorbable calcium for both human and animal consumption (EGGNOVO). Eggshell Calcium Supplement^TM^ (irRAWsistible) is another ES-based health product developed for the pet industry. Finally, Cluster Buster^®^ is advertised as an eco-friendly lightweight powder made from egg shell to capture flies. Insects sink in this patented powder (POWDER TRAP^®^).

## Intellectual Landscape of Eggshell Waste Applications

For this review, the Google patent research engine was utilized to identify global patents pertaining to ES in the last century (starting from 1920), and was searched using “eggshell” as a keyword. We observed that the annual number of ES patents was relatively stable and increased slowly over many decades; however, an exponential growth in patent numbers was observed during the last two decades (Figure 2). Overall, 673 patents pertaining to ES were granted in the last century. Of these patents, 536 (80%) were issued in the last two decades. The large leap in the last two decades was mainly due to a remarkable increase in the number of patents originating from Asian countries including China, Korea, Japan, and Taiwan (Figure 3). Interestingly, around 40% (257 patents) of these 673 patents were granted between 2015 and 2020 and address different biotechnological applications of ES. Around 40% (99 patents) of the patents published between 2015 and 2020 represent biotechnological applications of ES, while about 60% (158 patents) describe engineering technologies for the screening, separation and processing of ES. Recent advances in engineering technology patents are discussed in detail in the accompanying article (Ahmed et al., 2021). The main countries of origin of biotechnological patents were China (75.5%), Korea (9.3%), Taiwan (3.9), and the United States (3.1%; Figure 4). The dramatic increase in the number of patents that diversify the applications of ES reflects the interests of investors and researchers to exploit eggshell waste generated by breaker plants. This review sheds light on the most recent patents published between 2015 and 2020 for different biotechnological applications of ES (Table 1).

**TABLE 1:** Roadmap for the review.

**1. INTRODUCTION**	**1.1 Eggshell function and composition 1.2 Egg market 1.3 Eggshell waste and disposal 1.4 Eggshell waste added-value applications 1.5 Potential markets for eggshell waste 1.6 Commercially-available eggshell-based products**		
**2. INTELLECTUAL LANDSCAPE of EGGSHELL WASTE APPLICATIONS**			
**3. BIOTECHNOLOGICAL APPLICATIONS of EGGSHELL**	**3.1 Biomedical technologies**	**3.1.1** Production of calcium Lactate	
		**3.1.2** Production of Calcium Phosphate	
		**3.1.3** Health-promoting products	**3.1.3.1** Human
			**3.1.3.2** Animal
	**3.2 Chemical technologies**	**3.2.1** Plant growth promoters (fertilizer applications)	
		**3.2.2** Food processing and production	
		**3.2.3** Active calcium source	
		**3.2.4** Active carbon source	
		**3.2.5** Soluble protein source	
		**3.2.6** Organic calcium source	
		**3.2.7** Ultrafine active CaCO_3_ source	
		**3.2.8** Biodiesel oil catalysis	
		**3.2.9** Eggshell as component of plastic	
	**3.3 Engineering technologies**	**3.3.1** Material Engineering	
		**3.3.2** Nanoparticle (NP) production	
	**3.4 Environmental technologies**	**3.4.1** Adsorption of heavy metals	
		**3.4.2** Adsorption of organic compound	
		**3.4.3** Adsorption of total nitrogen, fluoride, and phosphate	
		**3.4.4** Adsorption of soil pollutants	
		**3.4.5** Adsorption of radioactive metal	
		**3.4.6** Solubilization of sludge	
		**3.4.7** Production of biomass	
		**3.4.8** Magnetic ES adsorbent	
**4. RECENT RESEARCH ACTIVITIES of EGGSHELL**			
**5. CONCLUSION and PROSPECTS**			

## Patented Biotechnological Applications for Eggshell

### Biomedical Technologies

Eggshell can be used as a source of medical calcium tonic and food additives including calcium lactate, calcium propionate, calcium gluconate, calcium citrate, and calcium acetate (Ran et al., 2018).

#### Production of Calcium Lactate

Calcium Lactate is widely used in the food industry as a curing, flavoring, leavening, and antimicrobial additive (Huang et al., 2019), in addition to its properties as a stabilizer, seasoning, and calcium-nutrition intensifying agent (Chen et al., 2019). The conversion of ES calcium into calcium lactate improves its solubility and absorption. New methods to prepare calcium lactate from ES have been patented to overcome the problems related to traditional calcination and acid-base neutralization methods (Chen et al., 2019).

During the last 5 years, many inventions have proposed microbial fermentation methods to produce calcium lactate from ES (Ma et al., 2015; Shao, 2017; Chen et al., 2019; Huang et al., 2019; Ma and Zhao, 2019). One method uses enzymatic hydrolysis and bacterial fermentation to produce lactic acid from ES. Firstly, powdered ES is converted to liquid through steps of heating and pressure homogenization, followed by treatment with papain to hydrolyze protein impurities. Following enzymatic hydrolysis, glucose and a combination of *Lactobacillus acidophilus (L. acidophilus*) and *Lactobacillus bulgaricus (L. bulgaricus*) is added to ferment the enzymolyzed ES liquid, which produces organic calcium. The extraction rate is claimed to reach 86%, with 93% calcium lactate and 5% calcium gluconate (Shao, 2017). Another invention proposes treatment of ES liquid with a mixture of enzymes (alkaline protease, neutral protease, and papain) to hydrolyze the proteinaceous impurities. Enzymolyzed ES liquid is mixed with glucose, and fermentation is initiated with a high-yield lactic acid-producing bacterium (*Enterococcus mundtii*, *E. mundtii*). Calcium lactate is precipitated with calcium hydroxide, purified, and crystallized (Chen et al., 2019). Another patented claim proposes the production of lactic acid from ES without a protease treatment step. Fermentation is carried out by *E. mundtii* in the presence of glucose followed by purification and crystallization steps. The purity of the produced calcium lactate is 99.25%, resulting in 2.5 kg calcium lactate/kg ES (Ma et al., 2015). Another strategy uses four strains of lactic acid bacteria including *E. mundtii*, *Streptococcus thermophilus* (*S. thermophilus*), *Lactobacillus casei* (*L. casei*), and *L. Bulgaricus* to ferment ES in the presence of glucose, with a calcium lactate yield of 40 g/L and purity of 93% (Huang et al., 2019). Improvements for the ES fermentation strategy have utilized immobilized cell technology, with *E. mundtii* embedded in sodium alginate. This immobilization strategy results in improved bacterial cell concentration, decreased fermentation time, and increased bacterial tolerance to pH, temperature, and organic solvents (Ma and Zhao, 2019). Calcium lactate production using microbial fermentation is cost-effective, environmentally friendly, and eliminates the need for the power-consuming calcination process (Ma et al., 2015).

#### Production of Calcium Phosphate

Tri-calcium phosphate (β-TCP) and HAP are two forms of calcium phosphate that have been widely utilized as biocompatible materials (Lee et al., 2016; Lee and Kang, 2017). HAP is an essential mineral which makes up hard tissues in the human body, such as bones and teeth (Lee et al., 2016; Lee and Kang, 2018). Calcium phosphate can be produced from ES via calcination at 950–1,050°C, followed by mixing with isopropyl alcohol and phosphoric acid. HAP, TCP, or a combination of both, are produced depending on the molar ratio of calcinated ES to phosphoric acid. The resultant calcium phosphate is ball-milled and heat treated, for applications in fabrication of artificial bone constructs (Lee, 2018). Similarily, pulverized and calcinated ES can be utilized as a starting material in combination with phosphoric acid to create HAP and TCP (Quina et al., 2017). The crystal phase and particle shape of the produced calcium phosphate can be modulated by controlling the phosphoric acid concentration, final solution pH and reaction temperature (Lee et al., 2016; Lee and Kang, 2017). Alternatively, calcium phosphate-based material can be produced using nano-sized calcinated ES powder and phosphoric acid-ammonia solution (Lee and Kang, 2018).

#### Health-Related Products

##### Human

Powdered ES is a rich source of “biological” calcium for oral dietary supplements that are proposed to increase bone density and treat age-related bone loss. In one application, Manuka Honey and lemon are added to ES powder to improve taste and smell and to enrich the final nutritional value of the calcium supplement (Shagdar and Myagmar, 2017).

Eggshell is the basis for a composite wound dressing for skin ulcers. ES, in combination with shell from litchi, peanuts, broad beans, and sesame, is treated with an enzyme mixture (1% Protease, 5% amylase, and 2% pectase). The enzyme hydrolysate is steam sterilized, fermented using *Bacillus subtilis* (*B. subtilis*), and filtered to obtain an extract based on plant material and ES. Finally, the extract is mixed with chitosan and alginate and electrospun to form a composite wound dressing with low production cost, good degradability, easy absorption, and good air permeability (Ye et al., 2019).

Chelated calcium can be prepared via an ES crushing strategy, which involves ES cleaning with sodium hypochlorite followed by drying and pulverization steps. The pulverized ES is dissolved in 15% HCl, and adjusted to pH 4 using NaOH. Solubilized proteins constituents are precipitated with phosphorylated corn starch flocculant and removed using centrifugation or filtration. The clear supernatant is mixed with either glycine or yeast, to form glycine-chelated or yeast-chelated calcium with a purity of 98%, for use in health care products and as food additives (Yang and Shen, 2017).

Eggshell-derived activated CaCO_3_ possesses excellent dispersibility and affinity for use as an incremental filler. ES is crushed, sieved, and mixed with water and stearamide before freeze-drying. The resulting ES powder is combined with starch, calcium silicate, calcium sulfate whiskers, calcium carbonate, lanolin, lecithin, trisodium citrate, sodium bifluoride, and then dried to obtain a compound calcium carbonate powder. The final step involves spraying this powder evenly with stearic acid (Wang, 2015a).

Eggshell-derived skin products have applications for whitening, exfoliating and moisturizing applications. Regarding skin cleansing products, soft beads made of polypropylene and polyethylene have raised environmental and skin safety concerns; therefore, ES powder has utility as a replacement for soft beads as a facial cleanser (Zhang, 2018).

##### Animal

Eggshell has applications as a calcium source in animal feed. Washed ES can be dried at 70–75°C, and ground (Urlovskis et al., 2016). Laying hens require calcium particles in their diet to maintain high rates of egg production. ES is incorporated in the layer feed to improve the bird health, increase egg production and maintain high eggshell quality (Li, 2017). ES powder is also used as a component of lobster feed to replace more expensive calcium-containing raw materials. Dried ES is cleaned, crushed, and sieved, followed by wetting with water, to be mixed with lobster feed at a ratio of 1:10 to 1:20 (Jin, 2018).

### Chemical Technologies

#### Plant Growth Promoters (Fertilizer Applications)

Calcium plays a vital role in plant growth. However, most inorganic calcium salts, including CaCO_3_, have low solubility in water and therefore exhibit unsatisfactory efficiency of uptake. Five (5) patents comprising new methods that convert ES-derived CaCO_3_ to useful soil fertilizers with improved calcium solubility have been issued recently (Liu et al., 2015; Capoani, 2016; Kim et al., 2018; Zhao and Wang, 2019; Yang et al., 2019d). ES can be converted to liquid calcium acetate fertilizer via the reaction of pulverized ES with acetic acid. As calcium acetate is relatively neutral (0.2 M solution is pH 7.6), it does not acidify soil when used as an amendment (Zhao and Wang, 2019). Dry, ground ES can be mixed with peanut bran, livestock and poultry manure, organic waste, and sprayed with microbial inoculants to produce a safe, environmentally friendly, and efficient fertilizer (Liu et al., 2015). Similarily, ES waste contaminated with ESM, EW, and EY can be treated and dissolved to produce a natural liquid fertilizer with improved solubility. In this process, ES is crushed and mixed with protease and a mixture of organic acids (acetic, malic, lactic, succinic, and citric acids) to decompose and ferment the proteinaceous contents. The organic acids react with ES to produce water-soluble calcium (Kim et al., 2018). Another approach, using marine shell extract, ES powder, and fermentation microbial agents, produces a fertilizer supplement for apple orchard cultivation. A mixture of ground abalone, oyster, and shellfish shells with calcinated ES improves apple fruit tree productivity and prevents disease (Yang et al., 2019d). Another fertilizer, produced from poultry manure and ES, is economic, environmentally friendly, and possesses high calcium and nitrogen content (Capoani, 2016).

#### Food Processing and Production

Eggshell has a wide range of applications in the food industry and in food production. Calcium-supplemented noodles can be prepared with incorporation of cleaned, disinfected, and sieved ES powder, which are claimed to be tasty and possess high nutritional value (Xie, 2018; Zeng, 2019). Organic calcium salts possess higher solubility and bio-availability than ES-derived calcium carbonate. An invention describes a method of preparation of sausage which contains a mixture of calcium acetate – calcium citrate derived from ES powder. The ES is cleaned, dried and ground, and added to a solution containing acetic and citric acids. The acetate-citrate composite calcium precipitate is dried and incorporated into the process of making sausage. The product provides a meat foodstuff which is enriched with readily absorbed dietary calcium (Liu et al., 2018). A cupcake with higher nutritional value can be prepared with ES mixed with standard ingredients (Giango et al., 2017). Cleaned ES powder can be mixed with crushed rice and fruit peel to produce a delicious wine beverage with high nutritional value (Wang et al., 2016).

Calcium propionate is used in the food industry as a preservative due to its antimicrobial activity against molds, aerobic bacilli, and gram-negative bacteria. In addition, it is readily absorbed in the GI tract and can provide essential calcium. A patent discloses a method to produce calcium propionate from ES as raw material. ES is cleaned, crushed, and sieved to obtain fine powder. The formed powder is solubilized in propionic acid, filtered and dried to produce soluble white scaly calcium propionate crystals with a purity of 98.5% (Wang, 2015b).

#### Active Calcium Source

**FIGURE 5:**
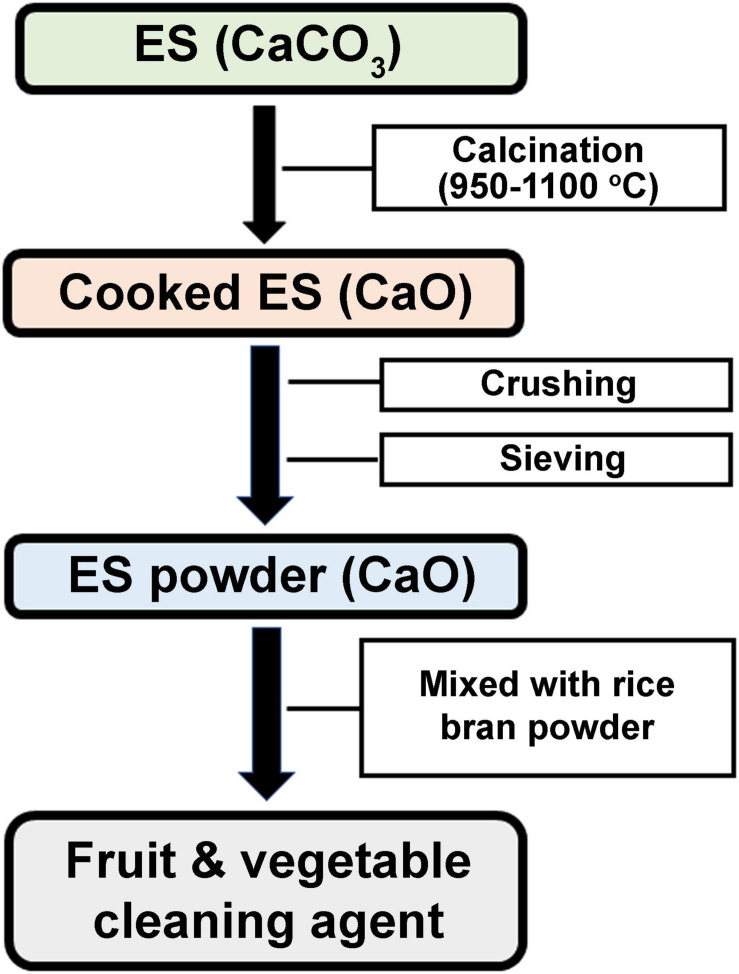
Schematic presentation of patent CN107057890A describing a method to prepare a cleaning agent for fruits and vegetables based on calcinated ES powder.

Calcium oxide is usually made by the thermal decomposition of calcium carbonate materials, such as limestone, seashells, or ES. Calcium hydroxide, also called slaked lime, Ca(OH)_2_, is obtained by the action of water on CaO. However, it is difficult to obtain highly active Ca(OH)_2_ using this strategy. A patent reports the use of pulverized ES to produce highly active porous Ca(OH)_2_. ES powder, calcinated at 650–750°C under negative pressure (−80 to −50 KPa), is reacted with air or oxygen to obtain highly porous CaO powder. The superfine CaO is enclosed in a CO_2_-free space with humidity of 90–95% to produce a highly active porous Ca(OH)_2_ with activity degree > 400, whiteness > 90, and surface area > 25 m^2^/g (Wang P. et al., 2019). Similarly, an ES-based porous CaO solid alkali can be produced using a simple and cost-effective temperature programmed ES roasting strategy. Pulverized ES is calcinated at 600–1,000°C to produce a solid alkali with abundant pores, high catalytic activity and a large specific surface area (Ding, 2018). Another method involves cleaning and disinfecting the fresh ES followed by stripping treatment and drying. Dried ES is crushed, sieved, and calcinated at 300–1,500°C to obtain calcinated ES powder (Yang, 2015). ES can be used as a natural ingredient in soap to overcome the problem of toxicity to human skin caused by conventional chemical compounds. The ES is dried at 100–200°C, pulverized and mixed with soap base. This natural soap product has cleaning, antibacterial and moisturizing abilities that render it suitable as a laundry or kitchen detergent, or as a personal beauty soap (Kang, 2016). As fruits and vegetables often have residues of pesticides and fertilizers, an invention utilizes ES to produce an abrasive scrubbing agent without the use of any additives. Pulverized ES is thermally decomposed into CaO through calcination at 950–1,100°C. The cooked ES (CaO) is then crushed, sieved, and mixed with fresh ES powder and bran powder to produce a very efficient, non-toxic, and harmless fruit and vegetable cleaning agent (Wang S. et al., 2017; Figure 5).

#### Active Carbon Source

Activated carbon has a rich pore structure and a high specific surface area. Adsorption based on activated carbon is widely used in the fields of raw material production for refining air and control of water pollution. A patented method uses powdered ES in combination with sucrose for the production of activated carbon. A mixture of ES powder with sucrose or glucose is calcinated at 800 or 1,100°C, respectively. Calcination is performed under an inert atmosphere and the product is then pickled using hydrochloric acid (HCl) to obtain activated carbon (Shi et al., 2019). The process of graphene synthesis consumes huge amounts of calcium carbonate. A disclosed patent describes the production of graphene using ES as a replacement for calcium carbonate, therefore reducing the industrial costs while carrying out mass production with simple equipment and operation. The process involves mixing ES with a reducing agent to obtain a powder which is then combusted under atmospheric conditions to produce a crude powder. Graphene is obtained by pickling the crude powder using HCl, followed by washing and filtration. The resulting graphene is a superior capacitor material. This approach requires a short reaction time and saves energy as it utilizes the energy released by the combustion reaction itself (Wang L. et al., 2019).

#### Soluble Protein Source

The organic matrix (3.5% of the ES) consists of proteins and proteoglycans which may possess valuable bioactive properties. Following removal of the outer cuticle and inner ESM with ethylenediaminetetraacetic acid, the cleaned ES powder is demineralized using acetic acid (2–23%), followed by centrifugation to separate the acid-insoluble organic matrix. The main advantage of this approach is the low concentration of the weak acid that does not degrade the organic matrix constituents. Moreover, the water solubility of acetic acid facilitates its removal in subsequent purification steps (Liu, 2017). Another invention discloses a method for co-purifying three abundant ES matrix proteins [ovocleidin-17 (OC-17), ovocleidin-116 (OC-116), and ovocalyxin-36 (OCX-36)] from ES by a combination of anionic and cationic exchange chromatography in phosphate buffer. This efficient co-purification of OC-17, OC-116, and OCX-36 achieves high purity (>90%; Ma et al., 2017).

#### Organic Calcium Source

Calcium citrate is a good source of calcium for dietary supplements, due to its high calcium content, high absorptivity, good solubility, low heavy metal content, and good ionic stability. Moreover, it does not interfere with the absorption of other critical ions including iron. A patent reports a method to produce calcium citrate from ES. Pre-heated ES powder is suspended in acetic acid to produce calcium acetate solution, which is then mixed with citric acid to produce calcium citrate. The invention also formulated a chewable tablet through the combination of calcium citrate with other additives including vitamin D (Zhu et al., 2016). Another invention describes the production of water-soluble ES calcium powder or liquid. ES is calcinated in an electric heating furnace at a temperature of 900 to 1,200°C. The purified calcinated ES is suspended in acetic acid (4–30%), converted into ionic organic calcium, and then dried and pulverized to produce water-soluble ES calcium acetate powder, which is rich in organic acids and 7 essential amino acids (Hyun, 2015).

#### Ultrafine Active CaCO_3_ Source

Calcium carbonate is widely used also in food production, medical fields, and chemical industries. An invention claims the preparation of an ultrafine active calcium carbonate from ES. After ESM removal, pulverized ES with particle sizes of 0.1–10 μm was mixed with water and a polysaccharide (starch, dextran, chitosan, or pectin), followed by freeze-drying. The edible polysaccharide is a hydrophilic modifier to activate ultrafine calcium carbonate (Ma et al., 2016).

#### Biodiesel Oil Catalysis

A patented invention describes a preparative method for modified ES biodiesel catalyst, involving drying and crushing steps to obtain ES powder, which is then immersed and roasted with sodium hydroxide and sodium nitrate to effectively increase its alkaline and acidic active groups. This greatly improves ES catalytic activity for preparation of biodiesel, with a high number of recycling cycles. Moreover, the conversion rate of catalytically produced biodiesel is high (Xiang et al., 2018).

#### Eggshell as Component of Plastic

A patented invention describes a method to prepare a resin composition comprising ES powder (5 to 50 parts) and high molecular polymer (100 parts). ES undergoes various processing steps including washing, ESM removing, drying, and pulverization. The high molecular polymer can be melt-kneaded with ES powder (150 μm) at a temperature below 200°C. The resin composition is described as an environmentally friendly material that can be used for packaging (Li, 2018). Another patented method mixes ES powder with polylactic acid to fabricate green packaging material (Li, 2020). Similarily, ES in combination with polylactic acid was patented for the production of packaging boxes with improved heat insulating ability (Li, 2019). In addition, ES powder has been patented as a raw material for the production of biodegradable plastic with improved degradability, flexibility and tensile strength (Wang and Song, 2015). Furthermore, a biopolymer blend containing engineered proteinaceous ES nanopowder can produce biopolymeric film with enhanced thermal stability, tensile strength and improved durability for the fabrication of food, waste, and biomedical packaging (Rangari and Tiimob, 2019).

### Engineering Technologies

#### Material Engineering

The grinding process to produce powdered materials in many fields, such as building materials, chemical industry, mining, and ceramics, grinding aids are used to increase the efficiency and output of the mill while maintaining the original material quality standards. A patent claims the use of ES powder as a grinding aid material, where ES is ground, sieved, moistened with water and mixed with the cement during milling. Use of the ES-based grinding aid reduced water consumption during milling and increased the product mechanical properties (Wang B. et al., 2019). ES can be also applied to architectural materials. A patented process claims the utilization of pulverized ES as a cement extender through mixing with ordinary sand, cement and water to produce a hollow block (Lagumen and Colmo, 2016). A similar patented process describes a strategy to produce porous building insulation material from ES and glass waste, which has low density, uniform pore size, high strength and good thermal insulation capacity. Briefly, pulverized ES as a foaming material is mixed with glass waste and heated at 800–1,000°C (Tian, 2015). Another patent describes the dissolution of ES in aqua regia/isopropyl alcohol solution to produce a CaCO_3_/CaO solution that is then mixed with 85% phosphoric acid (H_3_PO_4_) to form calcium phosphate glass precursor solution. This solution is heated at 800°C for three hrs to produce calcium phosphate glass that exhibits similar physical properties and better biocompatibility, as compared to traditional phosphate-based glass. The enhanced biocompatibility of ES-based glass is due to the presence of calcium (Lee and Kang, 2019). Furthermore, a patented process describes a sustainable approach to produce calcium silicate through the combination of pulverized ES, as a calcium source, with red ceramic chamote as the silicone source. The high temperature solid state reaction of this mixture at 1,000–1,100°C produced calcium silicate material with properties comparable to traditional calcium silicate materials that could be utilized as a ceramic material for thermal insulation (Guedes et al., 2016). Another patent reports a strategy to produce wood-plastic material using ES in combination with polypropylene, polyethylene grafted maleic anhydride, ethylene-vinyl acetate copolymer, nano-titanium dioxide, glass fiber, wood flour, silane coupling agent, and flame retardant. This wood-plastic material has excellent heat resistance, compression resistance and tensile resistance (Cao, 2018).

Interestingly, a disclosed patent describes the melting and binding of a homogenous mixture of polycaprolactone, polybutylene succinate, and ES powder at 120–190°C to produce a composite plastic material with superior tensile strength, modulus and elongation at break, that can be utilized in fabrication of toys, packaging, and in the electronics industry (Li et al., 2015). An ES powder-based synthetic paper preparation method involves the combination of powdered ES with a formulated polyurethane solution. ES as a biomass resource replaces the use of CaCO_3_ in the production of synthetic paper, and reduces the exploitation of natural resources and the environmental burden of mining (Wang D. et al., 2017). Finally, a dust-free chalk strategy involves the utilization of ES powder as a replacement for non-renewable gypsum resources. The dustless chalk described herein showed improved stability (Zhou et al., 2019).

#### Nanoparticle Production

**FIGURE 6:**
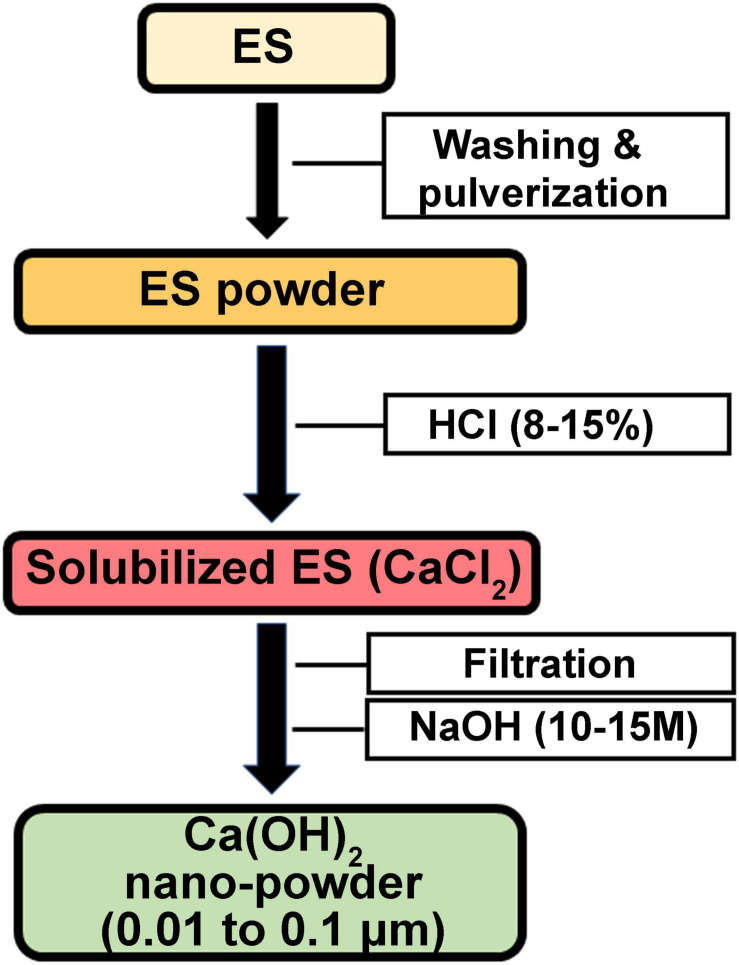
Schematic presentation of patent RO133975A0 describing a method to prepare NPs based on ES-derived Ca(OH)_2_.

**FIGURE 7:**
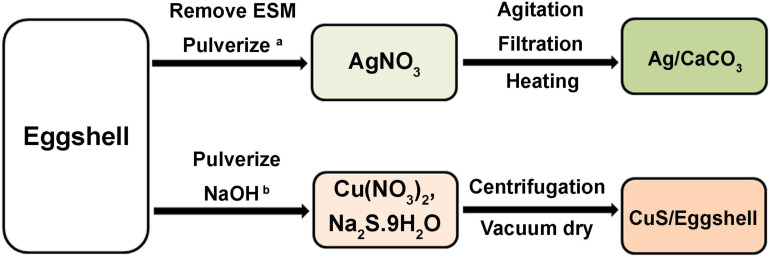
Schematic presentation showing the production of ES-based nanoparticles. a: Ag/Eggshell (CN106862585A), and b: CuS/Eggshell (CN109809466A).

The inherent pore structure of calcitic avian ES makes it an ideal natural scaffold for the production of nanoparticles (NPs; Yang et al., 2019c). CaCO_3_ constitutes about 95% of ES weight (Hincke et al., 2012; Neunzehn et al., 2015; Ahmed et al., 2019a), and has been used to produce Ca(OH)_2_ NPs. Pulverized ES is dissolved in HCl (8–15%), followed by filtration, and mixing with NaOH (10–15 M). The precipitated Ca(OH)_2_ is washed in water and oven dried at 100–150°C to produce Ca(OH)_2_ NPs having a CaO content of 96–99% and a particle size in the range of 0.01 to 0.1 μm. Ca(OH)_2_ NPs are used as an additive in inorganic binders to improve their reactivity and/or to develop high-performance mechanical properties (Panait et al., 2020; Figure 6). In addition, a patent disclosed a strategy to produce CaCO_3_-silver (Ag) NPs using ES as a template. ES is pulverized and loaded with Ag ions, followed by heating at 400–600°C. The resultant CaCO_3_-Ag NPs can be utilized for various applications including catalysis, tissue engineering, coatings, and production of antibacterial agents, pigments, and ceramics (Yang et al., 2017; Figure 7a). Another patented process demonstrates the production of copper sulfide (CuS) NPs using ES as a template. ES is pulverized and loaded with copper and sulfide ions to generate CuS NPs that exhibit antimicrobial activities against *Staphylococcus aureus* (*S. aureus*; Yang et al., 2019c; Figure 7b). Similarily, a patent claims the use of ES powder as a biological carrier that adsorbs copper (Cu) ions to form a complex copper nanomaterial. Cu NPs-loaded ES showed antibacterial activity against *Escherichia coli* (*E. Coli*) and *S. aureus* (Yang et al., 2019b).

Eggshell powder can serve as a template for the development of copper selenide-loaded ES NPs for catalytic degradation of organic pollutants in wastewater, such as 4-nitrophenol (Yang et al., 2019a). Alternatively, a patent disclosed a chemical process for the fabrication of iron (Fe)/ferrous sulfide (FeS)- loaded ES NPs, capable of degrading > 95% of 4-nitrophenol in wastewater (Yang et al., 2020). Similarly, pulverized ES loaded with Fe NPs can remove phosphorus from wastewater. Processing steps include treatment of ES with HCl, pulverization, addition of ferric salt and NaBH_4_/KBH_4_ solutions, drying to obtain the magnetically stable and oxidation-resistant nano adsorbent. The iron NP-loaded ES exhibited > 99% removal rate for phosphorus, and can be magnetically separated for easy recycling and regeneration (Xie et al., 2016).

### Environmental Technologies

Eggshell can also be used as a natural environmentally friendly biosorbent for heavy metals, dyes, organics, sulfonates, and fluorides (Baláž, 2014; Ahmed et al., 2019a), due to its abundance, low cost, and easy processing. As a potential adsorbent, ES combines some important characteristics including fast adsorption rate, superior adsorption capacity, and high selectivity for heavy metal ions in industrial wastewater (Tian, 2014). In addition, calcination of ES increases its pore size and surface area, for improved removal capacity for heavy metals and neutralization ability for strongly acidic wastewater (Park et al., 2007).

#### Adsorption of Heavy Metals

A patented process for treating wastewater uses ES calcinated at 850–900°C that showed an efficiency of 90% for removal of Cu, zinc (Zn), arsenic (As), and chromium (Cr; He et al., 2018). The chemical modification of calcinated ES (550°C) using silane coupling agent (KH-570) and tetraethyl orthosilicate leads to the formation of a promising adsorbent for Cr (VI) removal from industrial wastewater (Gao et al., 2017). Likewise, the aforementioned chemically-modified calcinated ES, in combination with carbonized rice straw and husk (produced at 400°C), binds heavy metal ions such as Cr (Chen, 2017). A granular composite adsorbent, combining the clay mineral sericite (Binder) and heat-treated ES (200–900°C), absorbs and removes the heavy metal lead (Pb II) from wastewater. Sericite as a binder serves not only as a spherical adsorbent but also has a synergistic effect to increase the adsorption rate of ES for heavy metals, while ES also serves as a neutralizer for acidic industrial wastewater (Choi, 2020). ES-modified activated carbon composite filter removes As from wastewater. Calcium-loaded activated carbon powder is prepared by soaking ground ES in acetic acid to obtain soluble calcium which is mixed with activated carbon. The resultant calcium-loaded activated carbon powder is mixed with ground ESM, polyethylene resin, and pore-forming agent (sodium chloride) to produce a composite filter element to remove As from drinking water (Sun, 2017b). The combination of the previously prepared calcium liquid with phosphoric acid-treated carbon produces calcium- and phosphate- loaded carbon. The modified carbon is mixed with zeolite and pulverized ESM to produce a composite capable of removing heavy metal ions and organic pollutants from lithium battery industrial wastewater with high efficiency (Sun, 2017a). Chemically modified ES powder (cobalt chloride, γ-aminobutyric acid, thyme and rhododendron extract, and isooctyl ferulate) efficiently removes cyanide (CN^–^), Cr(VI), Cu(II), Zn (II), and nickel (Ni II) from electroplating wastewater and may serve as an additive for rapid drying paint (Chen, 2018). Finally, a strategy to purify acid mine drainage (AMD) using a fixed bed adsorption process based on pulverized ES as an adsorbent has great potential to remove heavy metal including cadmium (Cd II), Pb (II), and Cu (II) from AMD. The discharge after adsorption meets water quality standards suitable for agricultural irrigation (Lu et al., 2016).

#### Adsorption of Organic Compounds

Avian ES is a potential adsorbent for removal of organic pollutants such as aromatic compounds that are present in many industrial wastewater effluents. The electrostatic nature of CaCO_3_ and its nanoporous structure make it a potential candidate for development of solid sorbents for the extraction of polycyclic aromatic hydrocarbons. Briefly, ES is treated with oxidizing agent, ground, and dissolved with HCl. Calcium carbonate crystals containing a monomeric Vateritic structure can be grown from a solution of calcium carbonate using various strategies (Nuhu et al., 2015). Alternatively, zinc oxide (ZnO)-loaded ES has been patented as an adsorbent material for organic matter and the photocatalytic decomposition of organic matter. ES is mixed sequentially with solutions of coupling agent, Zn ions, and alkali, and microwaved to form stable ZnO-loaded ES. The advantages of this invention are that the photocatalytic activity of ZnO is combined with the adsorption capacity of ESM to treat polluted water (Qiu et al., 2017).

#### Adsorption of Total Nitrogen, Fluoride, and Phosphate

Excessive nitrogen content is a key element in water pollution and eutrophication. Modified ES with a superior adsorption capacity for nitrogenous species to remove total nitrogen from wastewater can be prepared by combining pulverized ES with Fe (III) salt solution under the effect of heating (50–75°C; Li and Yan, 2020). Another method describes the use of pulverized ES to prepare a water-purifying fluoride-removal agent. After treatment with phosphoric acid or phosphate solution, followed by heating, ES is converted into a water purification agent that is superior to the commercially available defluoridination material (hydroxyphosphate; Pan and Tang, 2020). Recently, ES calcinated at 700°C (CES700) served as an efficient, low-cost, and eco-friendly adsorbent to recover phosphate ions from liquid effluents in batch or fixed-bed processes. CES700 achieved an adsorption capacity of about 39 mg PO_4_ equivalents_/_g at pH 8 (Santos et al., 2019). In the acidic range (pH < 6), an adsorbent material produced with iron oxyhydroxide and calcined eggshell (700°C) at a ratio of 1:0.5 (OFeCES.0.5) showed the best phosphate adsorption properties with a maximum adsorption capacity of 70 mg PO_4_ equivalents/g (Almeida et al., 2020).

#### Adsorption of Soil Pollutants

Eggshell adsorbent can be utilized for soil pollution control and agricultural remediation. Two patented processes claim the use of powdered ES as a soil conditioning agent for soil contaminated by heavy metals. ES powder is mixed with heavy metal-contaminated acidic farmland soil. ES absorbs heavy metals in the soil, and increases the soil pH, leading to a reduction in the absorption of heavy metals by crops and an increase in crop quality and yield (Lu et al., 2017, 2018).

#### Adsorption of Radioactive Metal

Uranium-containing polluted wastewater is highly injurious to animal/human health due to its radioactivity and chemical toxicity. A patent claims a utilization of powdered ES as a uranium adsorbent, with a uranium removal rate of 88% (Huang et al., 2017).

#### Solubilization of Sludge

Excess sludge is a byproduct of biological wastewater treatment that mainly comprises microorganisms and their cell wall glycan strands, which is resistant to biodegradation (Lee et al., 2019). A patent discloses a process to solubilize sludge through combining ultrasonication with modified ES. Pulverized ES is calcinated at 700–900°C and soaked in sulfuric acid solution, followed by a second calcination step to produce modified ES. Ultrasonication greatly increases the cracking rate of sludge cells and effectively reduces the cracking time for the remaining sludge. This process breaks through the moisture content bottleneck for sludge that is difficult to reduce below 60% after cracking. The resultant treated sludge has no odor and does not require external heating energy after cracking (Xiang et al., 2019). A magnetic slow-release carbon source can be prepared using municipal sludge and ES as raw materials. Pulverized ES is mixed with superparamagnetic ferrite material to produce magnetic hydrotalcite scaffold material, which effectively solves the problem of difficult recycling of the material after use. Hydrotalcite is a layered double hydroxide of general formula Mg_6_Al_2_CO_3_(OH)_16_⋅4(H_2_O). The magnetic hydrotalcite scaffold material is placed in the sludge fermentation broth, and the volatile acids that serve as a small molecule carbon source in the fermentation broth are adsorbed to obtain a magnetic slow-release carbon source (Yue et al., 2018).

#### Production of Biomass

**FIGURE 8:**
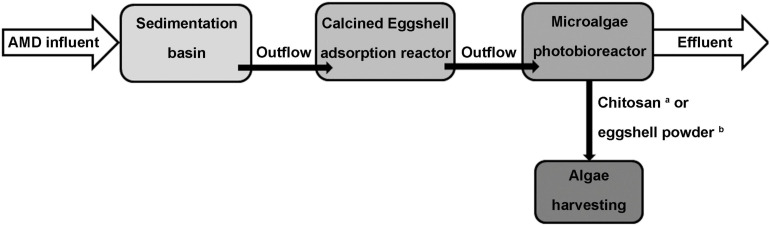
Schematic presentation showing the utilization of ES for the production of biomass. a: Acid Mine Drainage treatment using ES and microalgae hybrid system (KR101605096B), and b: harvesting microalgal biomass (KR101605199B1).

A patent describes a method to remove heavy metals present in the soil and simultaneously obtain biomass, using a combination of pulverized ES and microalgae. This hybrid system is capable of purifying AMD through acid neutralization and heavy metal adsorption activity, along with microbial contaminant removal. The heavy metal removal rate for the patented ES-microalgae combination was >95% for iron (Fe), Cu, Zn, manganese (Mn), and Cd. Moreover, the microalgae can be utilized as a biological source of new biomass for biodiesel production (Choi, 2016a; Figure 8). Interestingly, ES has also been patented as a nutrient for growth of microalgal biomass (Choi, 2016b; Figure 8).

#### Magnetic ES Adsorbent

A method for preparing a modified ES magnetic adsorbent to effectively remove heavy metals from wastewater is disclosed. Chemical and physical modifications are used to give ES its magnetic properties, which is cheap, excellent, stable, environmentally friendly and easy to recycle. Heavy metals are adsorbed on the magnetic adsorbent which is then collected by a magnet (Fan, 2016). Another invention uses the same magnetic ES-based adsorbent adapted to remove phosphorus from wastewater, with a removal efficiency of >99% (Fan et al., 2017).

Molecularly imprinted polymers (MIPs) are adsorbents for removal of environmental pollutants. Production of MIPs via the oil-in-water emulsion strategy requires the addition of surfactants that are harmful to the environment. Replacing surfactant with solid particles produces a Pickering emulsion, an emulsion that is stabilized by solid particles which adsorb onto the interface between the two phases. ES powder consists of solid particles that will stabilize a Pickering emulsion without any surface modification. Moreover, ES powder becomes magnetic with introduction of Fe_3_O_4_ NPs; their magnetic separation with an external magnetic field is straightforward. Magnetic imprinted microspheres produced in this manner exhibited selective recognition ability and adsorption capacity for the antibiotic erythromycin that is present in waste and river water (Zhu et al., 2019).

## Recent Research Activities of Eggshell

In addition to aforementioned patent-based survey of various ES biotechnological applications, recent research activities continue to be focused on developing high-value products from ES. Recent advances of ES in catalysis and mechanochemistry are summarized in a review paper (Baláž et al., 2021). To improve the poor bioavailability of calcium (Ahmed et al., 2019a), ES can be processed into nano-scale particles that exhibit higher water absorption capability and lower zeta potential, compared to micro-scale ES powder (El-Shibiny et al., 2018). Ball milling of micro-scale ES using zirconia balls in the presence of acetone (wet milling) facilitates the production of ES NPs in the size range of 1–100 nm (Puspitasari et al., 2019).

Eggshell-derived material showed great potential as an electrode for batteries and supercapacitors. ES can be utilized as a cathode, while calcined ES can be used as an anode (Minakshi et al., 2019). ES powder was also evaluated as a corrosion inhibitor for stainless steel (SS, Type 904) in an H_2_SO_4_ environment, and reduced the corrosion rate of SS (904) with 99% efficiency. In this regard, ES powder has potential as a mixed-type inhibitor with a predominant cathodic effect (Sannia et al., 2019). Furthermore, micronized ES powder has been exploited as an inert supplement, since partial cement replacement using ES powder produced concrete with a higher compressive modulus (Jhatial et al., 2019). In another application, ES powder was assessed as a fire retardant to produce fire-resistant cotton fabrics. Treated fabric incorporating ES shows lower flammability than the untreated material (Tseghai et al., 2019).

There are a number of interesting recent biomedical applications of ES, which has been evaluated as a scaffold component for bone regeneration. ES particle-reinforced gelatin hydrogels displayed superior mechanical properties and enabled the differentiation of pre-osteoblasts with significant enhancement in mineralization (Wu et al., 2019). Similarily, ES and nanotextured ES (NTES, phosphoric acid-mediated) particles incorporated into chitosan-alginate co-polymer scaffolds exhibited improved physicochemical properties along with enhanced mesenchymal stem cells retention, viability, and differentiation (Calvert, 2019). Nanocomposite electrospun fibers fabricated from poly(lactic) acid and needle-like HAP NPs derived from ES showed improved thermal and mechanical properties along with numerous pores and rough edges suitable for osteoblast cell attachment (Apalangya et al., 2019). In addition, scaffolds fabricated via simultaneous electrospinning of poly (ε-caprolactone)/polyvinyl alcohol/carbon dot in combination with ES-derived calcium phosphate supported a superior osteogenic differentiation and proliferation rate for human buccal fat pad-derived stem cells (Shafiei et al., 2019). Further, the nanocomposites prepared by mixing (2,2,6,6-tetramethylpiperidin-1-yl)oxyl (TEMPO)-oxidized cellulose nanofibrils (TCNFs) with HAP derived from ES led to the formation of composite with superior mechanical properties and uniform distribution of HAP particles. The fabricated composites improved the viability of human bone-derived osteoblasts (Ingole et al., 2020). Moreover, a composite fabricated from chitosan combined with ES-derived HAP exhibited enhanced thermal stability and roughness and showed reduced cytotoxicity when tested against human osteosarcoma cells (Saos-2; Trakoolwannachai et al., 2019). Finally, ES-HAP has been evaluated in multiple clinical studies as a substitute material in guided bone regeneration in oral surgery and showed positive outcomes when used for both cyst and socket defect models (Opris et al., 2020).

## Conclusion and Prospects

**FIGURE 9:**
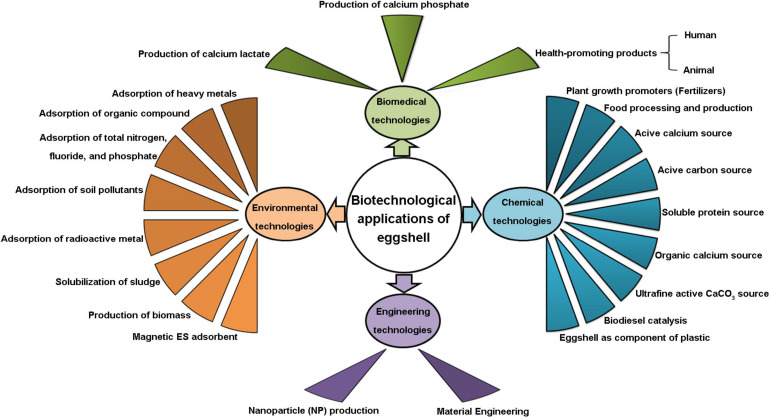
Schematic overview of biotechnological applications of eggshell.

In developed countries, almost one-third of shell eggs are diverted to egg breaking facilities to produce liquid eggs, leading to the generation of large amount of ES waste. ES disposal in landfill has a deleterious environmental impact. However, ES possesses characteristic biochemical, chemical and physical properties that make it an ideal raw biomaterial for various applications. This review has summarized the biotechnological applications of ES as a valuable bio-resource in terms of devices and methods patented between 2015 and 2020, which is a reasonable snapshot to capture its application scope. Creating value-added uses for ES waste is a sustainable waste management strategy, as it reduces the consumption of natural resources (limestone reserves) and reuses a valuable natural biomaterial while minimizing waste generation. In addition, such a strategy decreases the costs associated with disposal of ES waste. This overview of ES-based invention disclosures confirms the technological and economic feasibility of ES upscaling that attracts diverse industries to exploit its applications. Biotechnological applications of ES include patented inventions in the fields of biomedical, chemical, engineering, and environmental technologies. An overall schematic presentation of ES-based biotechnological applications is shown in Figure 9. The production of β-TCP and HAP for bone regeneration is a very promising biomedical application. ES as a source of ultrafine active CaCO_3_ and as an environmentally friendly packaging material are novel ES-based chemical technologies. Top-down strategies to produce ES-derived NPs represent a novel direction for engineering technologies. Innovative environmental applications of ES include the fabrication of magnetic ES adsorbents, adsorption of radioactive metals, and sludge solubilization. Engineering technologies for screening, separation, washing, sterilization, and processing are crucial steps to generate high-quality ES for value-added applications. Although such applications show enhanced economic value, ES byproducts are still relatively undervalued. In spite of the exponential increase in the number of ES – derived patents over the last decade, continued exploration of novel top-down approaches and further development of ES as a physical platform is essential to maximize its value.

## Author Contributions

TA, LW, MY, and MH approved of the manuscript’s content and warrant that this review manuscript is not under consideration for publication elsewhere. All coauthors have contributed in a significant way to the final format of the review.

## Conflict of Interest

The authors declare that the research was conducted in the absence of any commercial or financial relationships that could be construed as a potential conflict of interest.
